# Intricate and Cell Type-Specific Populations of Endogenous Circular DNA (eccDNA) in *Caenorhabditis elegans* and *Homo sapiens*

**DOI:** 10.1534/g3.117.300141

**Published:** 2017-08-11

**Authors:** Massa J. Shoura, Idan Gabdank, Loren Hansen, Jason Merker, Jason Gotlib, Stephen D. Levene, Andrew Z. Fire

**Affiliations:** * Department of Pathology, Stanford University School of Medicine, California 94305; † Department of Hematology, Stanford University School of Medicine, California 94305; ‡ Department of Bioengineering, University of Texas at Dallas, Richardson, Texas 75080; § Department of Biological Sciences, University of Texas at Dallas, Richardson, Texas 75080; ** Department of Physics, University of Texas at Dallas, Richardson, Texas 75080; †† Department of Genetics, Stanford University School of Medicine, California 94305

**Keywords:** *C. elegans*, circular DNAs, eccDNA, 3D genome architecture, circulome, mucin

## Abstract

Investigations aimed at defining the 3D configuration of eukaryotic chromosomes have consistently encountered an endogenous population of chromosome-derived circular genomic DNA, referred to as extrachromosomal circular DNA (eccDNA). While the production, distribution, and activities of eccDNAs remain understudied, eccDNA formation from specific regions of the linear genome has profound consequences on the regulatory and coding capabilities for these regions. Here, we define eccDNA distributions in *Caenorhabditis elegans* and in three human cell types, utilizing a set of DNA topology-dependent approaches for enrichment and characterization. The use of parallel biophysical, enzymatic, and informatic approaches provides a comprehensive profiling of eccDNA robust to isolation and analysis methodology. Results in human and nematode systems provide quantitative analysis of the eccDNA loci at both unique and repetitive regions. Our studies converge on and support a consistent picture, in which endogenous genomic DNA circles are present in normal physiological states, and in which the circles come from both coding and noncoding genomic regions. Prominent among the coding regions generating DNA circles are several genes known to produce a diversity of protein isoforms, with mucin proteins and titin as specific examples.

Endogenous DNA circles derived from canonical linear chromosomal loci, known as eccDNA, were first detected in nuclear fractions of plant cells (wheat and tobacco) in the 1980s by electron microscopy (Kinoshita *et al.* 1985). Since then, eccDNAs have been detected in human cell lines (Kinoshita *et al.* 1985; Assum *et al.* 1989; Misra *et al.* 1989; Kuttler and Mai 2007; Cohen *et al.* 2008) and cells of various organisms (Gaubatz 1990). Accumulating levels of eccDNA have been observed in connection with developmental progression (Gaubatz 1990; Gaubatz and Cutler 1990; Gaubatz and Flores 1990), aging (Kinoshita *et al.* 1985; Gaubatz 1990; Gaubatz and Cutler 1990; Sinclair and Guarente 1997), and genome instability (Cohen and Lavi 1996). Differences in eccDNA copy number and/or expression suggest that eccDNAs can contribute to genomic variation and mosaicism in different tissues, expanding the diversity in coding and regulatory capacity of eukaryotic genomes and transcriptomes.

A subset of eccDNA elements are associated with malignancies and drug-resistant tumors in a wide variety of cancers, such as circularized oncogenes and drug-resistance factors (“Double minutes”), which are capable of driving events in oncogenesis (Carroll *et al.* 1988; Albertson *et al.* 2003; Snijders *et al.* 2003). Beyond the known oncogene and mobile elements, multiple genomically unstable phenotypes are associated with an accumulation of eccDNAs (Gaubatz 1990; Cohen *et al.* 1997, 2003, 2008; Sinclair and Guarente 1997; Cohen and Segal 2009), including an observed rise in eccDNA levels in cells treated with carcinogens and in fibroblasts from patients suffering from Fanconi’s anemia.(Cohen *et al.* 1997) Moreover, deletions of genomic DNA segments in a circular form can occur in programmed processes such as RAG-dependent V(D)J recombination at the immunoglobulin and T-cell receptor loci in vertebrates. It should be noted that somatic deletions are generally investigated only when there have been tissue samples, resources, and a prior reason to expect a specific phenotype and/or locus to be associated with DNA rearrangement; therefore, there has been little opportunity to assess the level and scope of DNA deletions and corresponding eccDNAs in diseased or healthy cells. Even when they might be of interest, circular DNA elements would often be unrecognized or lost in whole-genome studies that depend on existing tools. Thus, eccDNA remains a relatively unexplored component of the eukaryotic genome (Cesare and Reddel 2010; Cohen and Segal 2009; Dilley *et al.* 2016).

Mechanistic features of eccDNA formation and metabolism similarly remain a mystery. Despite tremendous progress in our understanding of the synthesis, maintenance, and repair of eukaryotic linear genomes, not much is known about the fate of deleted/excised circular pieces of the genome. In some cases, there is clear evidence for retention of these circular DNAs (such as double-minute elements in cancer cells and telomeric circles (Carroll *et al.* 1988; Dilley *et al.* 2016; Li *et al.* 2017)). More recently, a study demonstrated that deleted circular DNA elements can be transcribed to produce dsRNAs, further contributing to small RNA-mediated genome reorganization (via piRNAs) in *Paramecium* (Allen *et al.* 2017). With the diversity of potential roles for eccDNA and eccDNA formation, studies focused on eccDNA provide a unique window into our understanding of the dynamic genome.

Several recent studies have combined high-throughput sequencing with protocols designed toward enrichment of eccDNA (Shibata *et al.* 2012; Moller *et al.* 2015; Kumar *et al.* 2017). These studies (in mouse tissues and yeast) have provided intriguing clues with respect to eccDNA sequence distributions. However, to obtain a comprehensive picture of eccDNA, it is critical to apply diverse methods that minimize DNA sequence- or structure-dependent biases (Kumar *et. al*. 2017). To this end, we used parallel methods to maximize the robustness of circular DNA isolation and sequencing. This analysis provides genome-wide “circulome” maps of a whole organism (*Caenorhabditis elegans*) and both healthy and diseased human tissues. We show that (i) genomic circular DNA repertoire is a function of cell type and state, (ii) eccDNA-mediated excision events are evident in both normal and diseased backgrounds, and (iii) a subset of eccDNAs map to several coding regions known to produce a diversity of protein isoforms.

## Materials and Methods

### C. elegans strains and maintenance

*C. elegans* were grown at 16° (unless specified) on agar plates with nematode growth medium seeded with *Escherichia coli* strain OP50 (Brenner 1974). Some strains were provided by the CGC, which is funded by the NIH Office of Research Infrastructure Programs (P40 OD010440). Strains used are: wild-type animals, VC2010 (PD1074), a clonal derivative of Brenner’s original *C. elegans* strain N2 (Brenner 1974), *glp-1*(*e2141ts*) (Austin and Kimble 1987; Yochem and Greenwald 1989), and fem-3(q20gf) (strain JK816) (Barton *et al.* 1987).

### Spermatocyte isolation

Sperm eccDNA was isolated from a *fem-3*(*q20gf*) mutant strain (Barton *et al.* 1987); this mutation converts a hermaphrodite to a sperm-only-producing strain. Sperm were isolated from a synchronized adult population of *fem-3*(*q20gf*) at the permissive temperature according to Gent *et al.* (2009). Briefly, after multiple washes in M9 buffer (to remove bacterial contamination), the animals were diced with a razor blade in a glass dish under the microscope. The mixture of released spermatogenic cells and carcasses was filtered through a double layer of 10 µm Nitex blotting cloth (Wildlife Supply) and washed three times in M9 before flash freezing in liquid nitrogen. A fraction of the sample was further fractionated by centrifugation for 20 min at (21,130 × *g*), the pellet was discarded, and the supernatant (containing smaller spermatocytes) flash frozen in liquid N_2_.

### Isolation of somatic tissue

A predominantly somatic tissue sample was isolated from a *glp-1*(*e2141ts*) mutant strain (Austin and Kimble 1987; Yochem and Greenwald 1989); *glp-1* encode!s a Notch signaling protein that induces the formation of a germline in *C. elegans*. When shifted to a nonpermissive temperature at the L1 larval stage, *glp-1*(*e2141ts*) animals produce animals with a full soma but with germline that is reduced by > 99%.

### C. elegans genomic DNA isolation

To prepare eccDNA and control genomic DNA, pellets of whole animals (or sperm pellets) were incubated with occasional gentle mixing in 450 µl of “Worm lysis buffer” (0.1 M Tris, 0.1 M NaCl, 50 mM EDTA, and 1% SDS, pH = 8.5) and 20 μl of 20 mg/ml Proteinase K (Roche) for 1.5 hr at 62° (< 25 µl pellet per sample). Standard procedures were used for DNA isolation. Briefly, after an NaCl precipitation step to remove debris (180 µl of saturated NaCl, spinning at 21,130 × *g* for 15 min, retention of supernatant), nucleic acids were ethanol-precipitated (1 ml of −20° ethanol), centrifuged at 4° for 30 min, pellets washed in 75% ethanol (room temperature), and resuspended in 100 μl TE (10 mM Tris-HCl and 1 mM EDTA, pH =8.0), and treated with RNAse A (Roche 2 µl of a 5 mg/ml stock) for 1 hr at 37°. After the addition of ammonium acetate to a final concentration of 1 M, DNA was purified by phenol–chloroform extraction and ethanol precipitation, followed by a 75% ethanol wash. Genomic DNA pellets were resuspended in TE and stored at −20°.

### Fibroblast and granulocyte DNA isolation

For comparison of fibroblast and granulocyte eccDNA profiles, DNA from a previous whole-genome sequencing study of a male individual (Merker *et al.* 2013) was used. Fibroblast cells were derived from a punch biopsy of healthy skin, while granulocytes (present at high levels due to myelofibrosis) were isolated from blood. The subject (Merker *et al.* 2013) was counseled and consented under a research protocol approved by the Stanford University Administrative Panel for the Protection of Human Subjects (Merker *et al.* 2013). DNA was extracted using the Gentra Puregene Cell Kit (QIAGEN, Valencia, CA) according to the manufacturer’s protocol.

### eccDNA enrichment

#### Cesium chloride-ethidium bromide (CsCl-EB) density gradient centrifugation:

**Figure 1:**
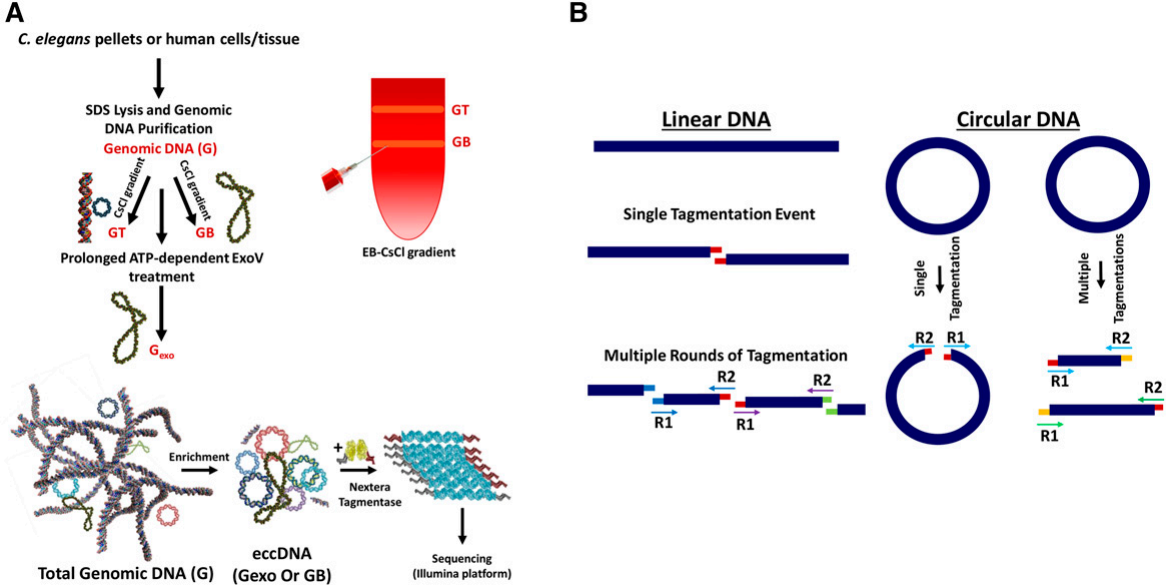
Workflow. (A) Genomic DNA is isolated from the organism/tissue of interest. Tissue is homogenized and treated with sodium dodecyl sulfate (SDS) and proteinase K. To enrich for circular DNAs, total genomic DNA (G) is treated with exonuclease V (exoV) (Palas and Kushner 1990) to produce G_exo_ or banded in a cesium chloride (CsCl) gradient to separate G into GT and GB (Grossman *et al.* 1974). GT is the upper band of the gradient and includes linear DNAs and nicked circular DNAs. GB, the bottom band, consists of covalently closed-circular DNAs. After enrichment for circular DNA with either method (or both), eccDNA (extrachromosomal circular DNA) is minimally sheared by attenuated treatment with Nextera tagmentase. (B) Transposition creates a 9-bp sequence duplication flanking the transposon insertion site. Tn5 randomly binds and cuts DNA, leaving a staggered, nine-nucleotide single-stranded overhang. DNA on either side of the cut is filled by DNA polymerase in the first polymerase chain reaction (PCR) cycle, thereby creating 9-bp duplications flanking the genomic DNA sequence. Matching overhangs in the figure have matching colors. Also, paired reads (R1 and R2, indicated by arrows) share the same color. If a circular DNA molecule gets cut only once by Tn5, paired-end sequencing will reveal a unique 9-bp duplication at the beginning of each read (designated by colored overhangs), thereby providing a bioinformatic mark for circular DNAs.

High-molecular weight genomic DNA was mixed into 2.0 ml of a CsCl solution having a density of 1.55 g/ml. The sample was subjected to centrifugation at 500,237 × *g* for 2.5 hr in a S120-VT vertical rotor (Thermo Scientific). As a reference, a plasmid DNA sample was run in parallel in a separate centrifuge tube. In the absence of exonuclease V (exoV) treatment, a distinct band, corresponding to sheared linear nuclear DNA (along with nicked/relaxed DNA circles), was visible under ultraviolet light. The plasmid DNA control sample showed two distinct bands corresponding to the nicked and linear (top) and covalently closed plasmid (bottom). A hypodermic needle was used to carefully isolate the fraction of interest with the closed circular plasmid band used as an indicator of the approximate location of the invisible eccDNA band (Figure 1A). Ethidium was removed from the isolated bands by extraction with CsCl/TE-saturated 1-butanol. Samples were dialyzed for 2 d against TE buffer (10 mM Tris-Cl and 1 mM Na_2_EDTA) at 4°.

#### ExoV treatment:

For enzymatic removal of linear DNA, 200 ng of genomic DNA was treated with 400 U/µg exoV (NEB) over 3 d in 50 mM potassium acetate, 20 mM Tris-acetate, 10 mM magnesium acetate, and 1 mM DTT, pH 7.9, in the presence of ATP and 100 µg/ml ampicillin (to limit bacterial contamination). After each round of exoV treatment, the reaction was heat inactivated at 70° for 30 min. A similar protocol was followed for human genomic DNA treatment, except for the duration of the reaction (5 d). For human eccDNA experiments, three different exoV to DNA ratios were used ranging from 500 to 1000 U/µg (Figure 1A).

### Library preparation and low-input Nextera protocol

To generate fragmented genomic DNA libraries with appropriate linkers, 1 ng of DNA was treated with 1.5 µl of Nextera XT tagmentase (Illumina) at 37° for 30 min with gentle shaking. For eccDNA libraries with DNA input < 1 ng, the eccDNA sample was treated with 0.5 µl of Nextera XT tagmentase at 37° for 30 min. In experiments where an enrichment for singly tagmented circles was sought, the tagmentation reaction was attenuated, with 0.5 µl of tagmentase incubated with the DNA at 37° (without shaking) for only 3 min. A minimal number of PCR cycles was chosen by monitoring the amplified DNA by gel electrophoresis after varying numbers of PCR rounds, ensuring libraries are prepared from PCR reactions in which the amplified DNA was still undergoing amplification. We find that amplification up to 10–12 cycles of PCR is sufficient for library production.

### Plasmids and synthetic DNA mini-circles

As a template for generating reference DNA circles, we used a plasmid with two directly repeated loxP sites cloned in the backbone of the generic vector pGEM5Zf(+) (Shoura *et al.* 2012; Shoura and Levene 2014). The resulting plasmid, pCS2DloxPzero (Shoura *et al.* 2012), allows insertion of arbitrary spacer sequences (gBlocks, IDT) between the two loxP sites by linearizing the plasmid with both *Not*I and *Pst*I (NEB). Using pCS2DloxPZero as a vector, we inserted a 378 bp insert sequence between the two loxP sites, resulting in plasmid pCS2DloxP378. Upon treating pCS2DloxP378 with Cre recombinase [(purified in-house according to Martin *et al.* (2002) and Gelato *et al.* (2005)], a 412 bp circle is produced (378 bp + one loxP site; 34 bp), along with the 3034 bp parent plasmid.

### Bioinformatic analysis

Bowtie2 (version 2.2.25) was used align the paired-end reads to the nematode (ce10) or human (hg38) reference genomes, respectively. Mapped reads were deduplicated using Picard. Unique reads were sorted and indexed using samtools (1.2.). To analyze sequences that cannot be mapped uniquely, a separate positioning approach was used. This approach uses both unique and repeated k-mer sequences (Li *et al.* 2014) to characterize individual k-mer/read counts and positions (python scripts available on request). Reads were divided into categories as follows. Unique Chromosomal: these reads represent the number of different read pair start/stop positional combinations for which both reads are uniquely and unambiguously mapped the reference genome (consistent with Bowtie algorithms). Locally repeated reads or “focal repeats”: defined as sequence that occur multiple times in the genome but for which all occurrences are confined to a single chromosome in a limited range of base pair distance (chosen as 300 kb for this study). Dispersed repeats: repeated reads that are distributed beyond this limited range or on multiple chromosomes. Intrachromosomal repeats: defined as reads that map to multiple sites on a single chromosome where the sites are separated by long distances (in this case, above the arbitrary cutoff of 300 kb). We used the Dfam databases (Hubley *et al.* 2016) to annotate the repetitive elements found in the eccDNA fractions.

### Enrichment analysis

Each eccDNA sample was sequenced in parallel to a control genomic DNA sample (G) from the same biological specimen. Read counts for each genomic interval (bins of 1 kb for *C. elegans* and 25 kb for human) were obtained from each eccDNA sample and its paired control. To identify exemplary enriched regions in the eccDNA fractions, we used a minimum enrichment value of fourfold, required that a greater than fourfold enrichment be robust to expected (binomial) stochastic variation in read counts, and used a two-tailed Bonferroni-corrected false discovery rate of 0.05.

### Data availability

The authors state that all data necessary for confirming the conclusions presented in the article are represented fully within the article. Sequencing data will be publicly available on ENCODE (the Encyclopedia of DNA Elements) under project file set ENCSR984FML.

## Results

### Circulome-Seq as a hybrid biophysical-biochemical-bioinformatic method to characterize genomic circular DNAs

The approach described here entails two independent and effective separations of linear and circular DNA: (i) a subtractive biochemical, enrichment-based method, multiple rounds of extensive digestion with exonuclease V (removing the vast majority of linear DNA) (Palas and Kushner 1990) and (ii) a biophysical purification method, centrifugation in CsCl/ethidium-bromide gradients, which provide topological separation of circular forms from linear DNA (Grossman *et al.* 1974). Applying one or both of these separations, we show a substantial enrichment for circular DNAs evidenced by enrichment for the circular mitochondrial DNA, which serves as an internal control (see Figure 1). In both approaches, we avoid: (a) DNA-purification methods that include denaturation and subsequent renaturation steps (*e.g.*, based on alkaline lysis and neutralization), as these steps enrich for repetitive linear DNA fragments along with circles (Wetmur and Davidson 1968); (b) digestion of genomic DNA with restriction enzymes (Moller *et al.* 2015) or selection of a specific DNA size range through size-exclusion columns (Shibata *et al.* 2012), which naturally biases enrichment in favor of small eccDNAs; and (c) “rolling circle” amplification of input DNAs to increase circular DNA copy number before subsequent processing, which will decrease the fidelity of eccDNA molecules and potentially introduce various artifacts (Fire and Xu 1995; Fujii *et al.* 2014; Kumar *et al.* 2017). Notably, we find that essentially identical populations of circular species are obtained using either method (i) or method (ii) (see Supplemental Material, Figure S1, A and B).

Following enrichment by procedure (i) or (ii), or both (i) and (ii), we find that eccDNAs can be simultaneously fragmented and tagged via a Tn5 transposition-based fragmentation and tagging system (Nextera tagmentase) (Caruccio 2011; Reznikoff 2008). The use of tagmentation has the advantage of allowing us to work with very low levels of input material (<1 ng of eccDNA). An additional advantage of using Nextera fragmentation, in particular for small circles, is the duplication of a 9-nt segment of the target sequence on opposing sides of each transposon insertion (Gerstein *et al.* 2010). This feature provides a precise bioinformatic signature for the presence of singly tagmented circular DNAs in a sequenced eccDNA pool (Figure 1B and Table 1). Our ability to capture circles in the protocol was confirmed with control circular substrates (3000- and 400-bp DNA circles) (Figure S2). Extensive analysis of *C. elegans* eccDNA shows that eccDNA enrichment is captured in a quantitatively reproducible manner (Figure S1). Confirming the specific role of exoV in eccDNA enrichment, we note that eccDNA is unenriched when ATP was omitted from the ATP-dependent exoV reactions (Figure S3).

**Table 1:** Capturing singly tagmented eccDNA circles

Sample Name	Fraction of 9-bp Duplication Incidents Relative to Total Captured Incidents (%)	Fold Enrichment of Singly Tagmented Circles in eccDNA Fractions Over the Corresponding Total Genomic DNA
Gexo	0.84	311.1
GB	0.74	277.8
Spermexo	0.22	27.5
glp-1exo	0.4	181.8
G	0.003	—
SpermG	0.008	—
glp-1G	0.002	—

eccDNA, extrachromosomal circular DNA.

### eccDNA distribution in C. elegans somatic cells and germline

**Figure 2:**
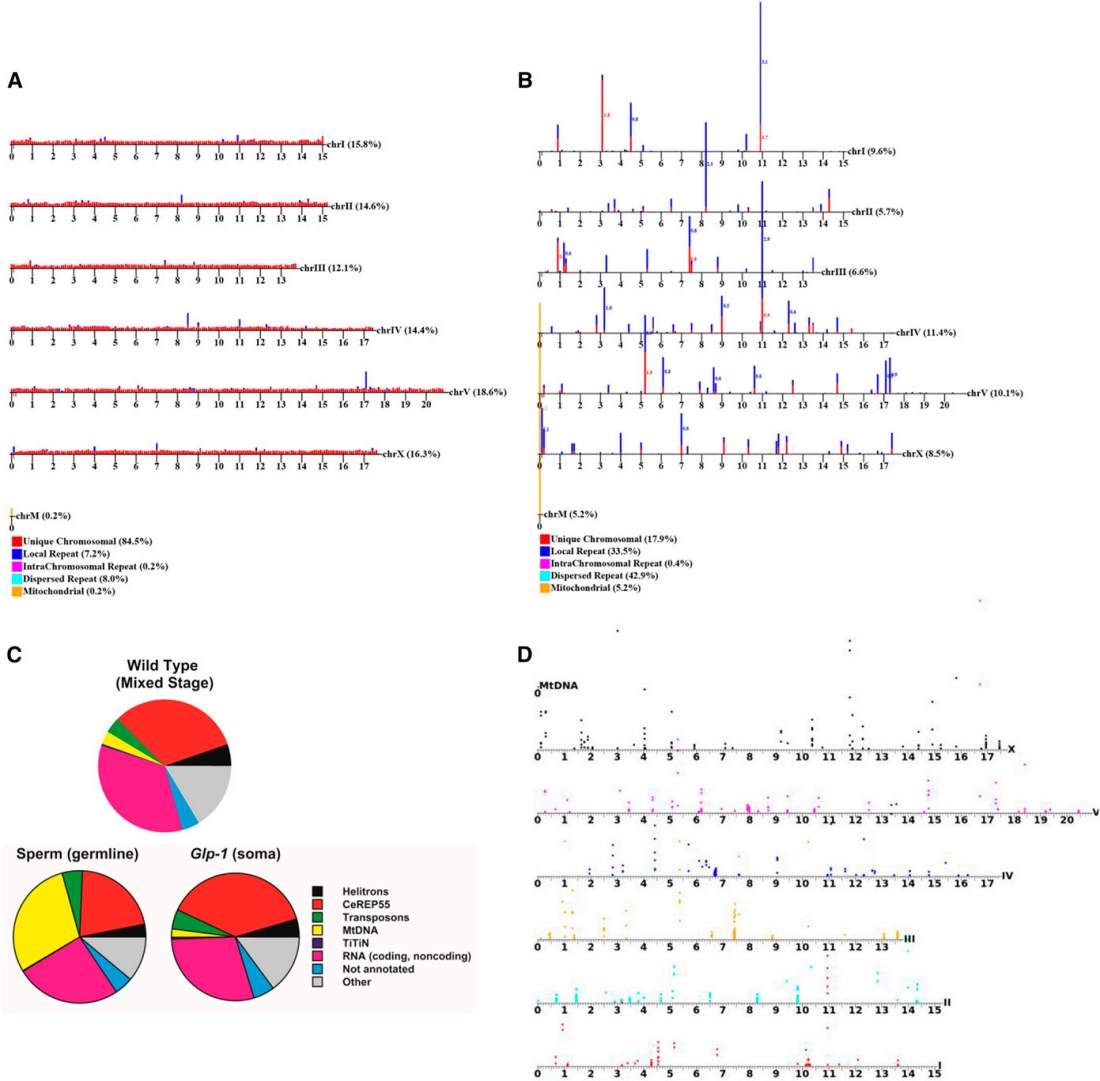
Data analysis. (A and B) are chromosomal maps of aligned reads in total genomic DNA (G) and eccDNA (G_exo_, extrachromosomal circular DNA), respectively. Reads are categorized as: unique, local repeats, intrachromosomal repeats, and dispersed repeats. The graphs show unique reads and focal repeats only, as dispersed repeats cannot be mapped to one location. (C) Whole-genome distribution of sequence classes in eccDNA fractions from WT animals, *C. elegans* sperm, and animals lacking germline cells. (D) Our methodology applied to *glp-1* animals (somatic adults). This map shows uniquely mapped areas on each chromosome that are significantly enriched in the circular pool {1-kbp intervals with enrichment assessed through Bayes maximum-likelihood [minimum of twofold enrichment with a default false discovery rate of 0.05/(2*number of genes)]}. This plot shows only reads that map uniquely to the genome. Position of the colored circle on the *y*-axis for each interval is proportional to the degree of enrichment.

**Figure 3:**
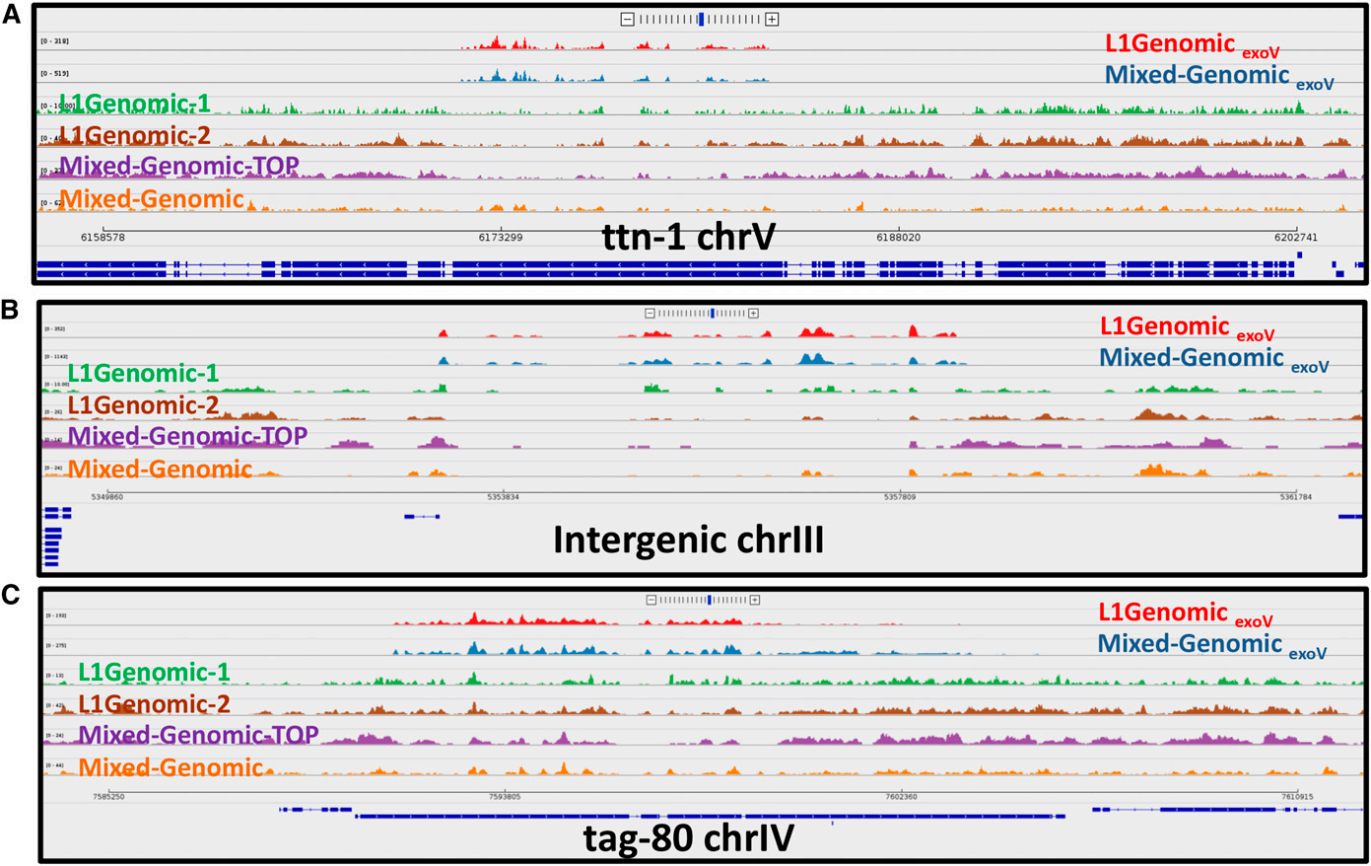
Sequence coverage of three eccDNA (extrachromosomal circular DNA)-enriched regions in different *C. elegans* populations. (A–C) show three distinct regions in the genome where eccDNA is generated. Red: exonuclease V (exoV)-treated DNA from synchronized young larvae, L1; blue: exoV-treated DNA from a mixed-stage population. Green, brown, and orange tracks are untreated genomic DNAs (L1 Genomic-1 and L1 Genomic-2 are independent biological replicas). (A) Hyper-enriched eccDNAs isolated from exoV-treated *C. elegans* genomic DNA map precisely to a coding exon of the titin gene in the eccDNA pool. Unique mapping of untreated genomic DNA from the top band of a cesium chloride-ethidium bromide gradient is shown in purple. (B and C) show similar profiles for eccDNAs corresponding to an intergenic repeat and the *tag-80* gene, respectively.

To examine eccDNA distributions in a complex whole organism, we carried out circular DNA isolation and sequencing on eccDNA preparations from *C. elegans*. We investigated mixed stage *C. elegans* (whole animals), synchronized young larvae (L1 stage), synchronized germline-deficient adults (*glp-1* mutants, predominantly somatic tissue), and *C. elegans* sperm cells (Figure S4. These analyses identified a diverse population of eccDNAs including segments from coding exons, transposons, repetitive regions, telomeric sequences, and other unannotated genomic locations (Figure 2 and Figure 3). A substantial portion of eccDNA sequences originate from helitrons (a class of mobile elements known to transpose via a circular intermediate) (Kapitonov and Jurka 2007), cut-and-paste transposons, and exons (for a complete list of locations of eccDNAs and enrichment levels, see File S1). Of these families, we were most surprised to observe circles directly derived from coding regions. Among the most abundant species are *ttn-1*, *plg-1*, *srap-1*, *clec-80*, *clec-223*, *frm-1*, *arrd-27*, *Y46B2A.3*, and *tag-80* genes (*ttn-1*, *Y46B2A.3*, and *tag-80* are the *C. elegans* orthologs of titin, mucin-1, and piccolo/aczonin, respectively.) The *ttn-1* gene encodes a large protein that is essential in muscle function in *C. elegans*. Interestingly, the specific titin exon that is producing eccDNAs encodes an extended protein domain noted for its strong potential to form elastic structures of diverse lengths (Guo *et al.* 2012; Khan *et al.* 2016; Werfel *et al.* 2016).

### Characterizing eccDNA distributions in three different human tissues

**Figure 4:**
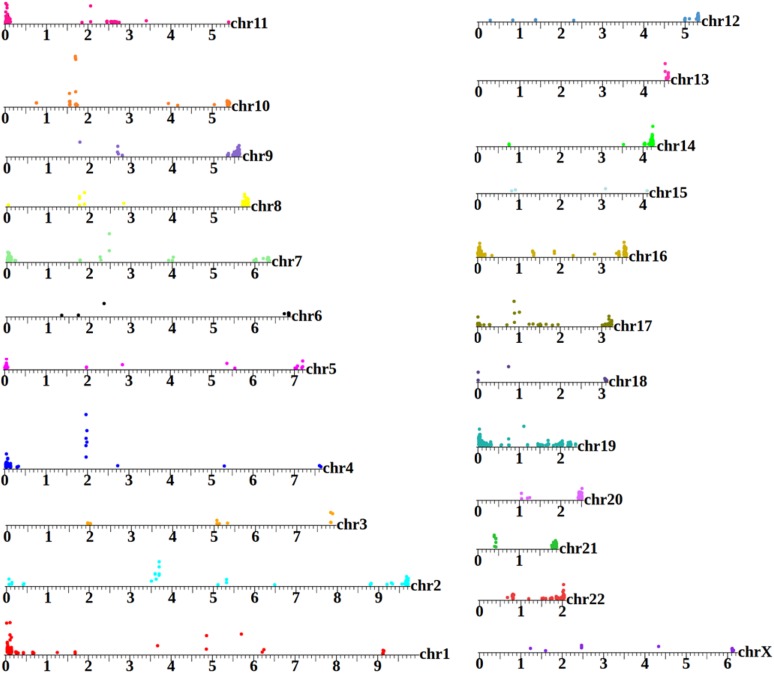
A human extrachromosomal circular DNA (eccDNA) map. GA12878 is the canonical cell line used by the National Institutes of Standards and Technology as a benchmark for high-throughput genome analysis (Zook *et al.* 2014). However, to date, published analysis of this sample to has been focused on the linear genome. This map shows areas on each chromosome that are significantly enriched in the circular pool (25-kbp intervals with enrichment assessed through Bayes maximum-likelihood). Position of the colored circle on the *y*-axis for each interval is proportional to the degree of enrichment. The eccDNA profile of each chromosome is distinctive, with enriched regions aligning to coding segments, repetitive, and subtelomeric sequences. This plot shows only reads that map uniquely to the genome.

To characterize eccDNA populations in human cells, we isolated eccDNA from human genomic DNA samples obtained from three sources: (i) a lymphoid cell line that has been subject to extensive sequence analysis and used as a standard for technical and software benchmarking in the genomics community (“Genome in a Bottle” cell line; GM12878) (Zook *et al.* 2014); (ii) neoplastic granulocytes from a patient with primary myelofibrosis, a subtype of myeloproliferative neoplasm; and (iii) a normal nontransformed primary fibroblast population from the same patient. This analysis showed extensive, but region-specific, eccDNA production (Figure 4). Overall, classes of sequences compared closely with those previously identified in the *C. elegans* genome, such as coding and noncoding segments along with focal and dispersed repetitive sequences. Moreover, we find that a significant portion of the GM12878 circulome maps to mucin genes (such as *MUC1*, *MUC2*, *MUC6*, and *MUC17*) encoding high-molecular weight proteins characterized by the presence of large amino acid tandem repeat sequences that show allelic size variation (Jia *et al.* 2010; Fowler *et al.* 2001; Linden *et al.* 2008; Walsh *et al.* 2013). A complete list of the eccDNA coordinates in GM12878 is presented in File S2.

**Figure 5:**
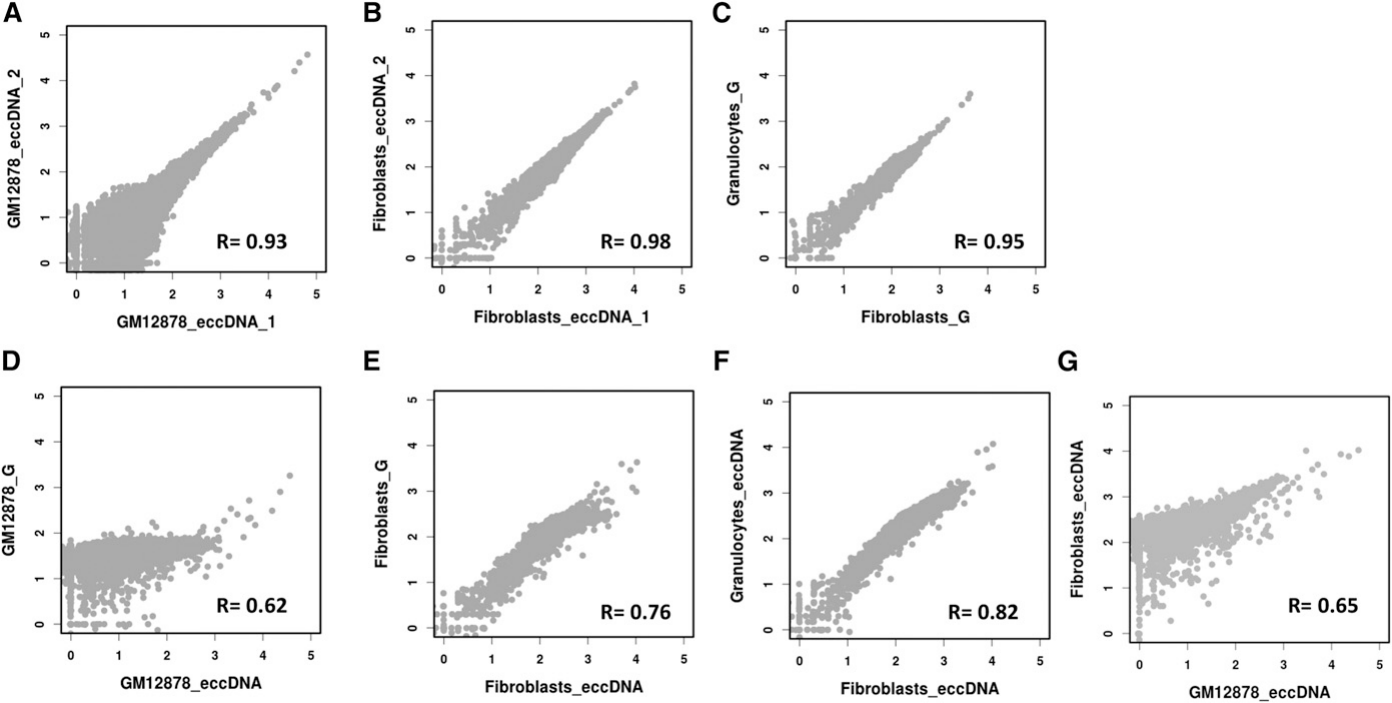
Analysis, reproducibility, and cell-type specificity of human extrachromosomal circular DNAs (eccDNAs). (A–G) showing log_10_ of read coverage for each chromosome with a bin size of 100 kbp. (A and B) show that eccDNA from the same tissue type is captured reproducibly. (C) Similar reproducibility is obtained for total genomic DNA (G) from different tissue types (normal fibroblasts *vs.* myeloproliferative neoplasm granulocytes from the same individual). Distinct differences are evident when eccDNAs are compared to their reference total genomic DNAs (D and E) as well as when eccDNAs from different cell types are compared (F and G).

To evaluate the sensitivity and robustness of the human assay and to assess whether eccDNA profiles are cell-specific, we compared circular DNA profiles between biological replicates of each cell type and between cell types. The eccDNA profiles obtained for each biological replicate pair are highly correlated, which indicates the reproducibility of the assay (Figure 5, A and B). Between the different cell types, we observe substantial differences, with cell state/type as a likely component in determining the diversity in the circulome. This holds true even when we compared eccDNAs in fibroblasts and granulocytes derived from the same donor (Figure 5) (Merker *et al.* 2013).

## Discussion

In summary, we present a rigorous approach for isolating and purifying endogenous circular DNAs from *C. elegans* and human tissues. We have identified thousands of eccDNA regions in the genomes of *C. elegans* and human cells. Interestingly, the identified eccDNA species are enriched for specific exons that encode multi-isoform proteins (*e.g.*, titin, mucins, and piccolo/aczonin). A main finding of this study is that different cell types harbor different repertoires of circular DNAs. It has been shown that eccDNA copy number can be modulated by chromatin remodeling machinery (Peng and Karpen 2007). Therefore, we speculate that the circulome of a cell is a function of the genome’s unique and tandemly repeated sequence elements, recombination hotspots, and potentially of open chromatin. Whatever the determinants that drive production of eccDNA circles from specific regions in specific cell types, the ramifications for genome activity and genetic diversity between cells is substantial.

### Comparison of eccDNA characterization methods

eccDNAs have mystified scientists for three decades, with the investigation of eccDNA phenomena reflecting diverse approaches to characterization, first at individual loci and more recently on a genome-wide scale. Several highly specific methods from the early characterization of individual loci provided definitive proof of eccDNAs as endogenous elements (Vinograd and Lebowitz 1996). In a remarkable set of studies starting in the 1990s, Cohen *et al.* (2003, 2008, 2010) and Cohen and Segal (2009) adapted two-dimensional (2D) gel electrophoresis for the detection and characterization of eccDNA. Each DNA population (supercoiled, open circular, linear single, and double-stranded), consisting of molecules of heterogeneous size, migrates as a separate arc, allowing simultaneous analysis of size range, amount, and sequence content of both supercoiled and open circular eccDNA. While this method offers a rich protocol for the analysis of specific loci, it is low throughput. The rise of NGS allowed for a whole-genome characterization of any genomic feature of interest. Applied to eccDNA, Shibata *et al.* (2012) and Moller *et al.* (2015) offered additional insights into the distribution of eccDNA in mouse and yeast cells. Both of these studies relied on alkaline denaturation/renaturation and prolonged rolling circle amplification to enrich for circles and produce abundant molecular populations for capture and sequencing. While yielding a plentiful molecular population, these steps also have a substantial potential to enrich for repetitive DNAs and other molecules that might be favored but do not bear a circular topology (Wetmur and Davidson 1968; Fire and Xu 1995; Fujii *et al.* 2014). In addition, applying size-exclusion columns to purified eccDNA molecules (Shibata *et al.* 2012; Kumar *et al.* 2017) can be used to focus on specific subpopulations of the eccDNA pool, but will preclude a comprehensive analysis of these molecules.

In devising the biochemical and computational methods used for the study presented here, we have endeavored to build a framework in which the full suite of biophysical tools that have been used to characterize DNA topology can be augmented by NGS. The Circulome-Seq methods described herein utilize newer capture and sequencing technologies, allowing the use of small amounts of starting eccDNA while avoiding methods such as alkaline lysis, rolling circle replication, and size-exclusion chromatography. As the technical capabilities to characterize large molecular populations advance, opportunities for interpretation rely on bioinformatic analysis of both unique and repetitive components of the genome. Using a k-mer approach (Li *et al.* 2014) combined with careful partitioning between local and global repeats allows optimal assignment of eccDNA to locally duplicated regions such as titin and mucins (Figure S5). Using a capture tool (Tn5 transposase) that duplicates a significant sequence element (9 bp) allows for isolation-independent confirmation of circular topology for individual molecules. It is our hope that the results, methods, and analyses presented here will contribute a definitive foundation to the genome-wide understanding of eccDNA distribution, dynamics, and mechanistic processes.

## Supplementary Material

Supplemental material is available online at www.g3journal.org/lookup/suppl/doi:10.1534/g3.117.300141/-/DC1.
