# Supplementary material for: Intricate and Cell Type-Specific Populations of Endogenous Circular DNA (eccDNA) in Caenorhabditis elegans and Homo sapiens
Source: G3 (Bethesda). 2017 Aug 11;7(10):3295–303. doi: 10.1534/g3.117.300141 (PMC5633380; doi:10.1534/g3.117.300141)
Supplement: Supplementary file 1 [file 3295FigureS1.pptx]

## Slide 1
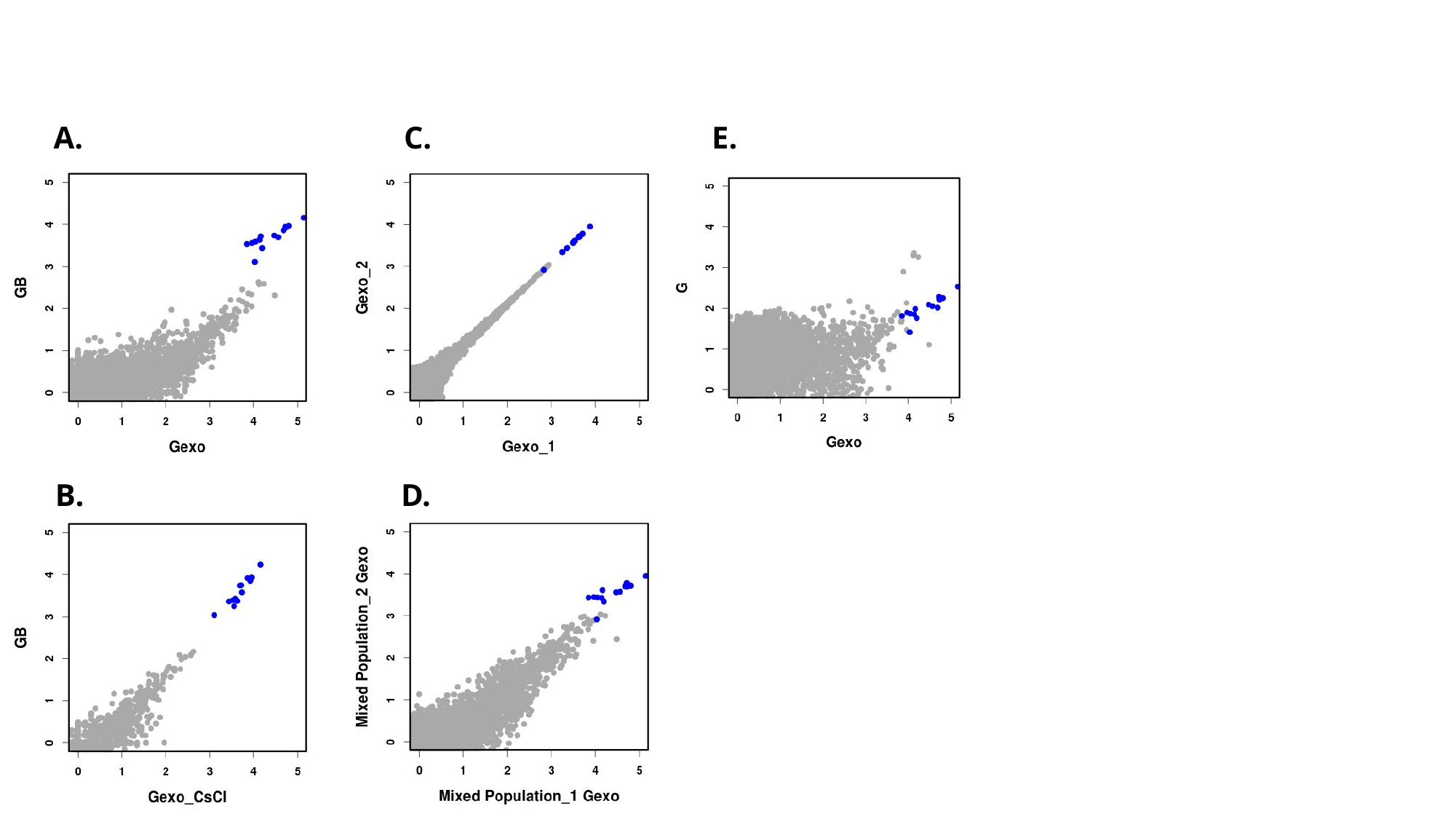

A.
C.
E.
B.
D.

## Slide 2
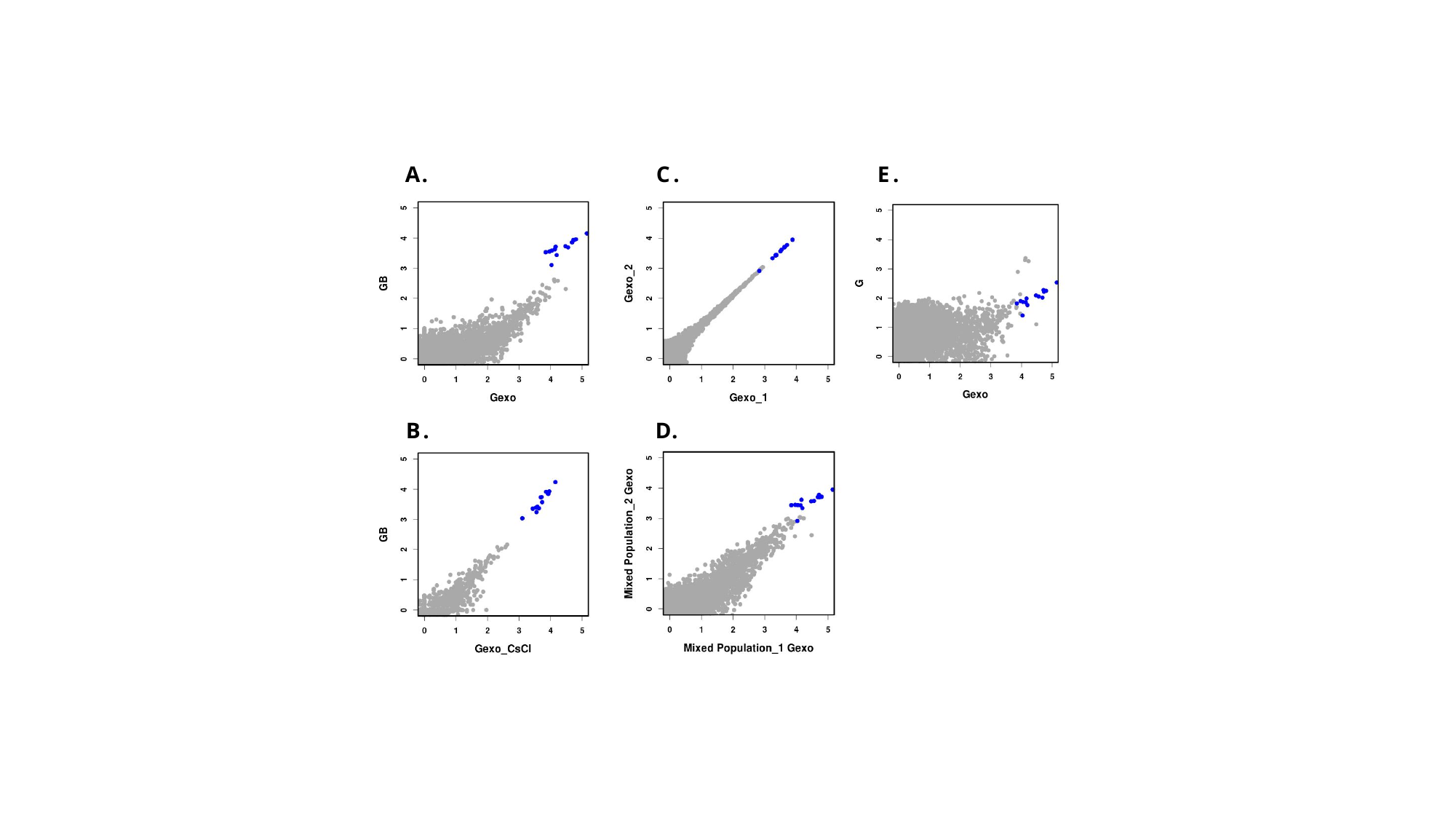

## Slide 3
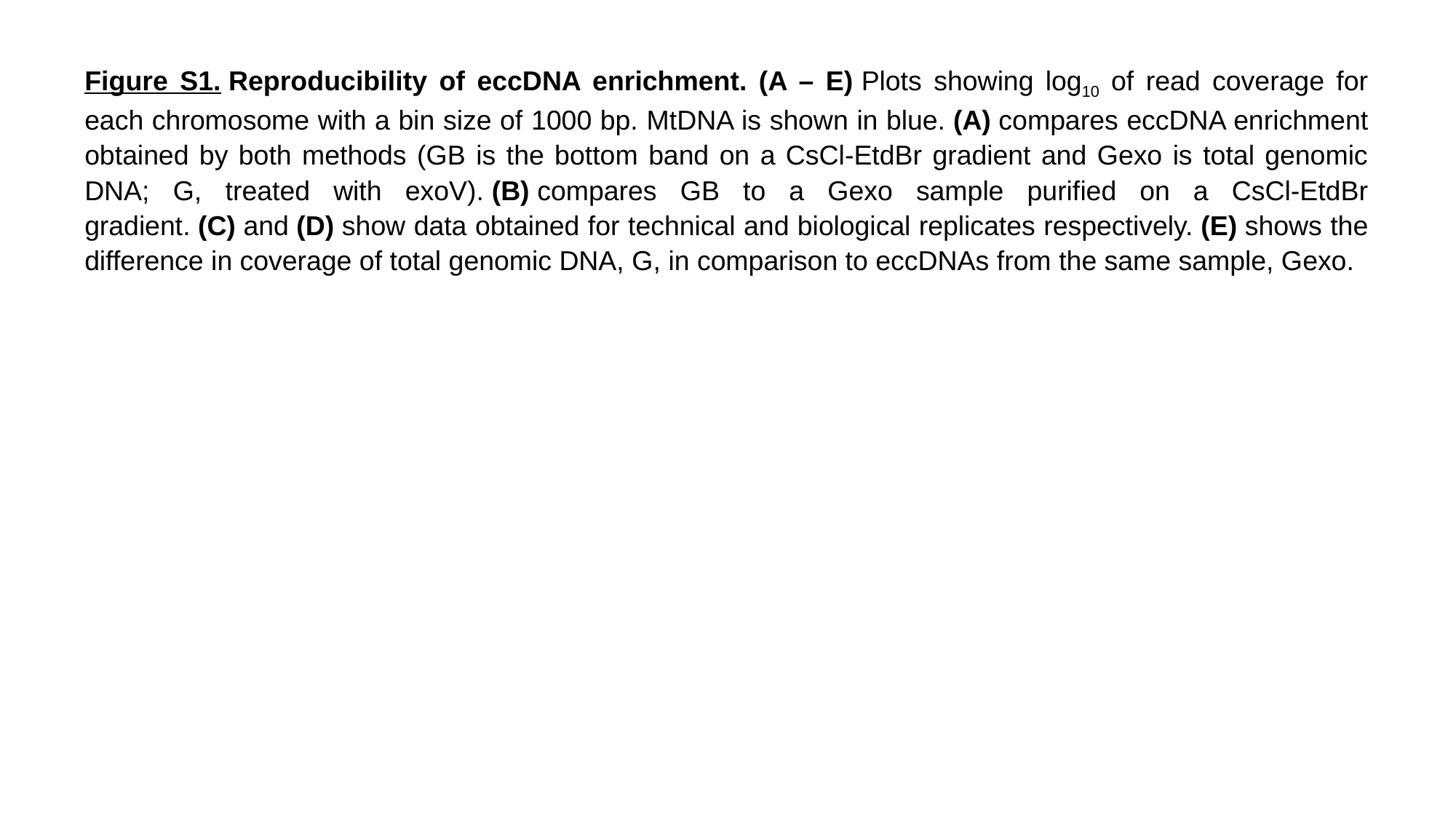

Figure S1. Reproducibility of eccDNA enrichment. (A – E) Plots showing log10 of read coverage for each chromosome with a bin size of 1000 bp. MtDNA is shown in blue. (A) compares eccDNA enrichment obtained by both methods (GB is the bottom band on a CsCl-EtdBr gradient and Gexo is total genomic DNA; G, treated with exoV). (B) compares GB to a Gexo sample purified on a CsCl-EtdBr gradient. (C) and (D) show data obtained for technical and biological replicates respectively. (E) shows the difference in coverage of total genomic DNA, G, in comparison to eccDNAs from the same sample, Gexo.
