# Supplementary material for: Intricate and Cell Type-Specific Populations of Endogenous Circular DNA (eccDNA) in Caenorhabditis elegans and Homo sapiens
Source: G3 (Bethesda). 2017 Aug 11;7(10):3295–303. doi: 10.1534/g3.117.300141 (PMC5633380; doi:10.1534/g3.117.300141)
Supplement: Supplementary file 2 [file 3295FigureS2.pptx]

## Slide 1
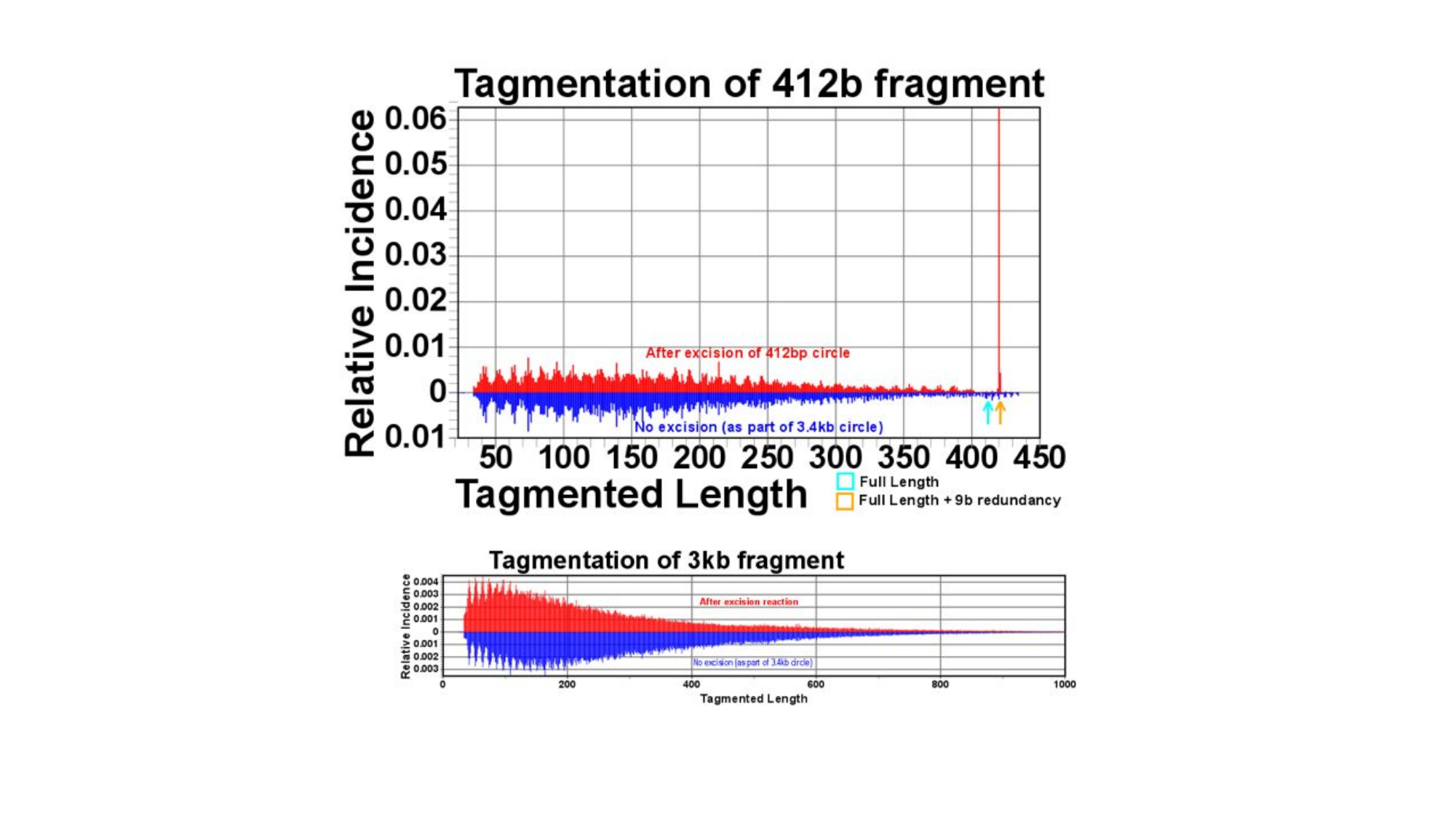

## Slide 2
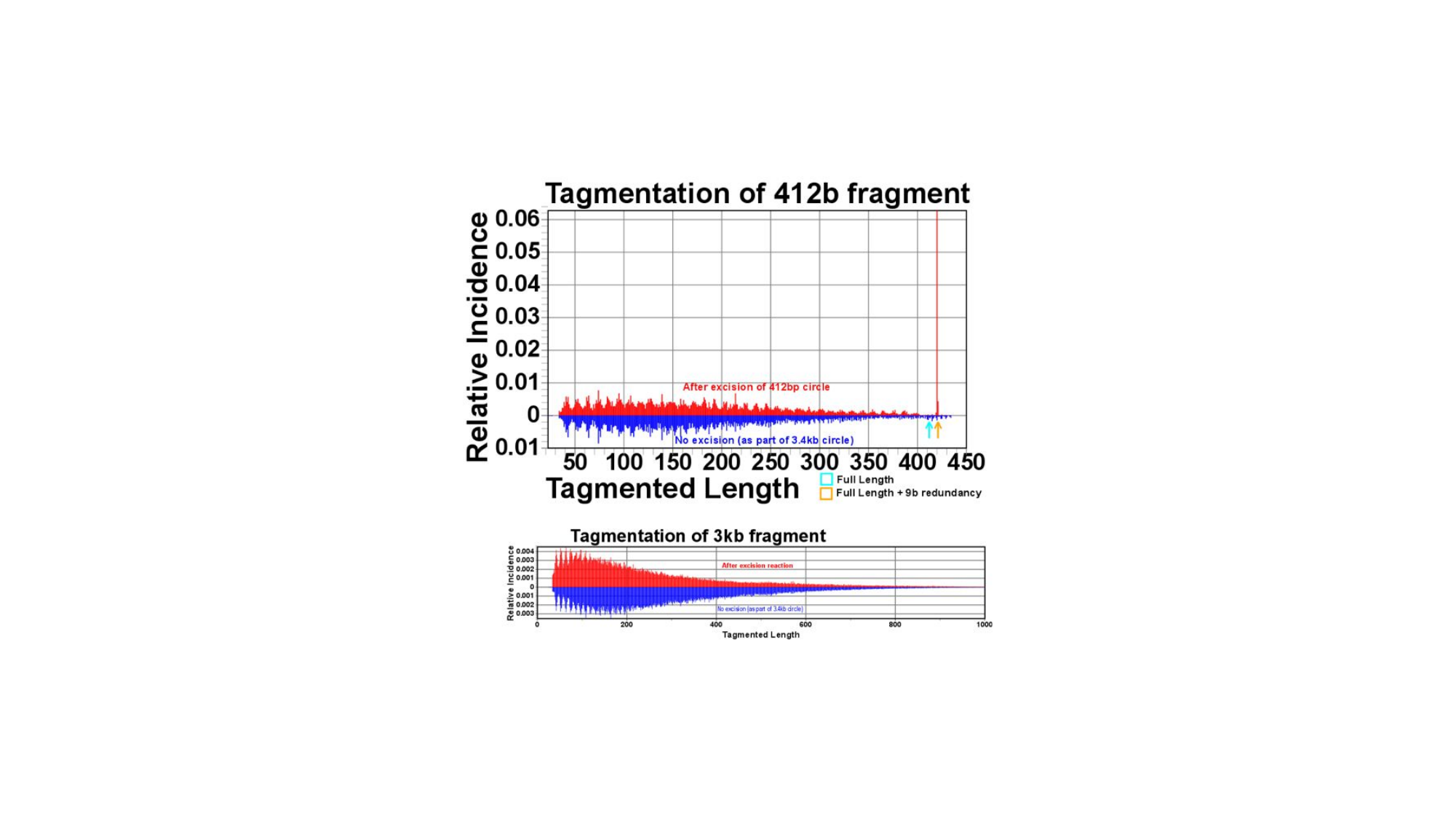

## Slide 3
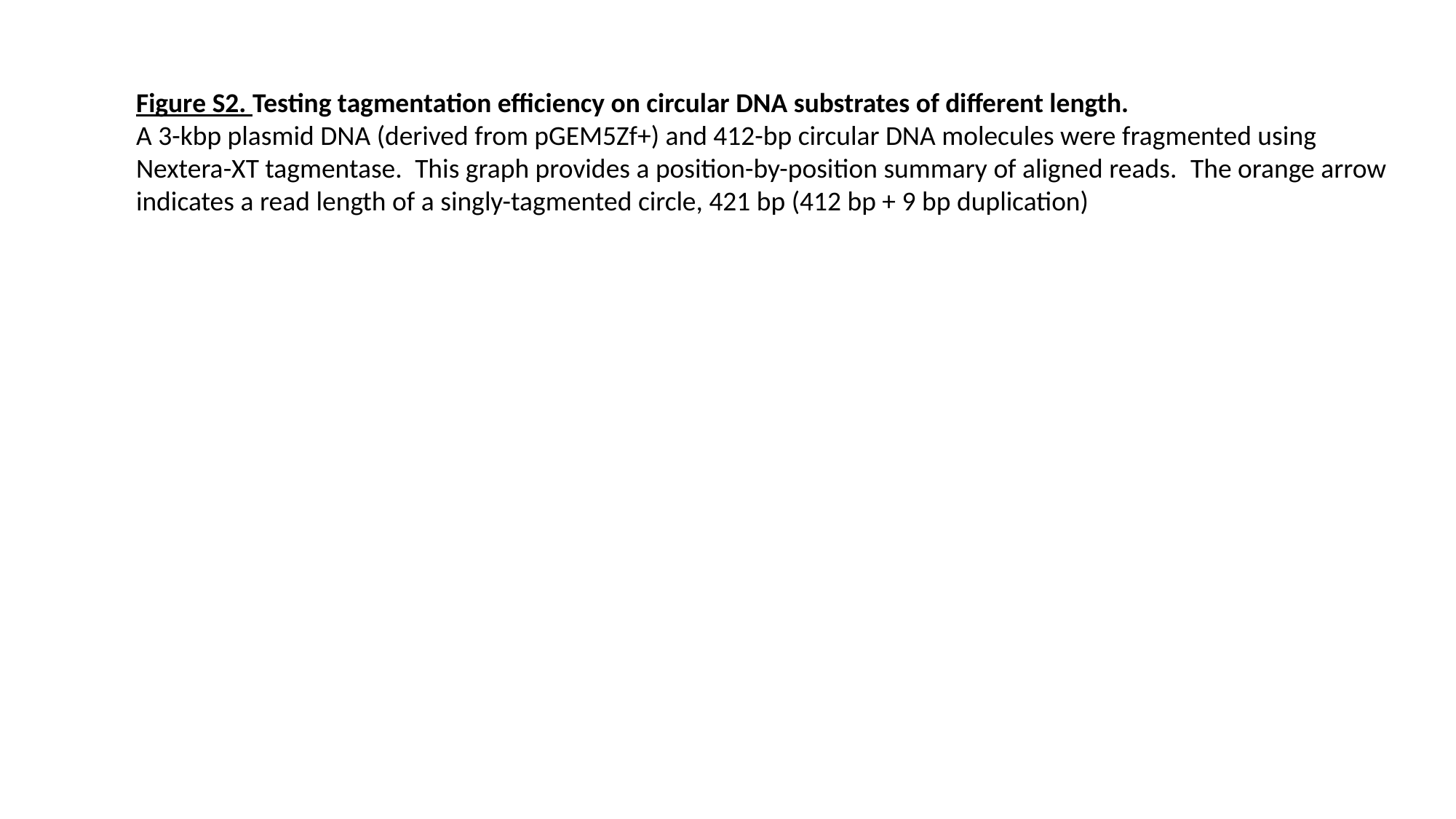

Figure S2. Testing tagmentation efficiency on circular DNA substrates of different length.
A 3-kbp plasmid DNA (derived from pGEM5Zf+) and 412-bp circular DNA molecules were fragmented using
Nextera-XT tagmentase.  This graph provides a position-by-position summary of aligned reads.  The orange arrow
indicates a read length of a singly-tagmented circle, 421 bp (412 bp + 9 bp duplication)
