# Supplementary material for: Intricate and Cell Type-Specific Populations of Endogenous Circular DNA (eccDNA) in Caenorhabditis elegans and Homo sapiens
Source: G3 (Bethesda). 2017 Aug 11;7(10):3295–303. doi: 10.1534/g3.117.300141 (PMC5633380; doi:10.1534/g3.117.300141)
Supplement: Supplementary file 3 [file 3295FigureS3.pptx]

## Slide 1
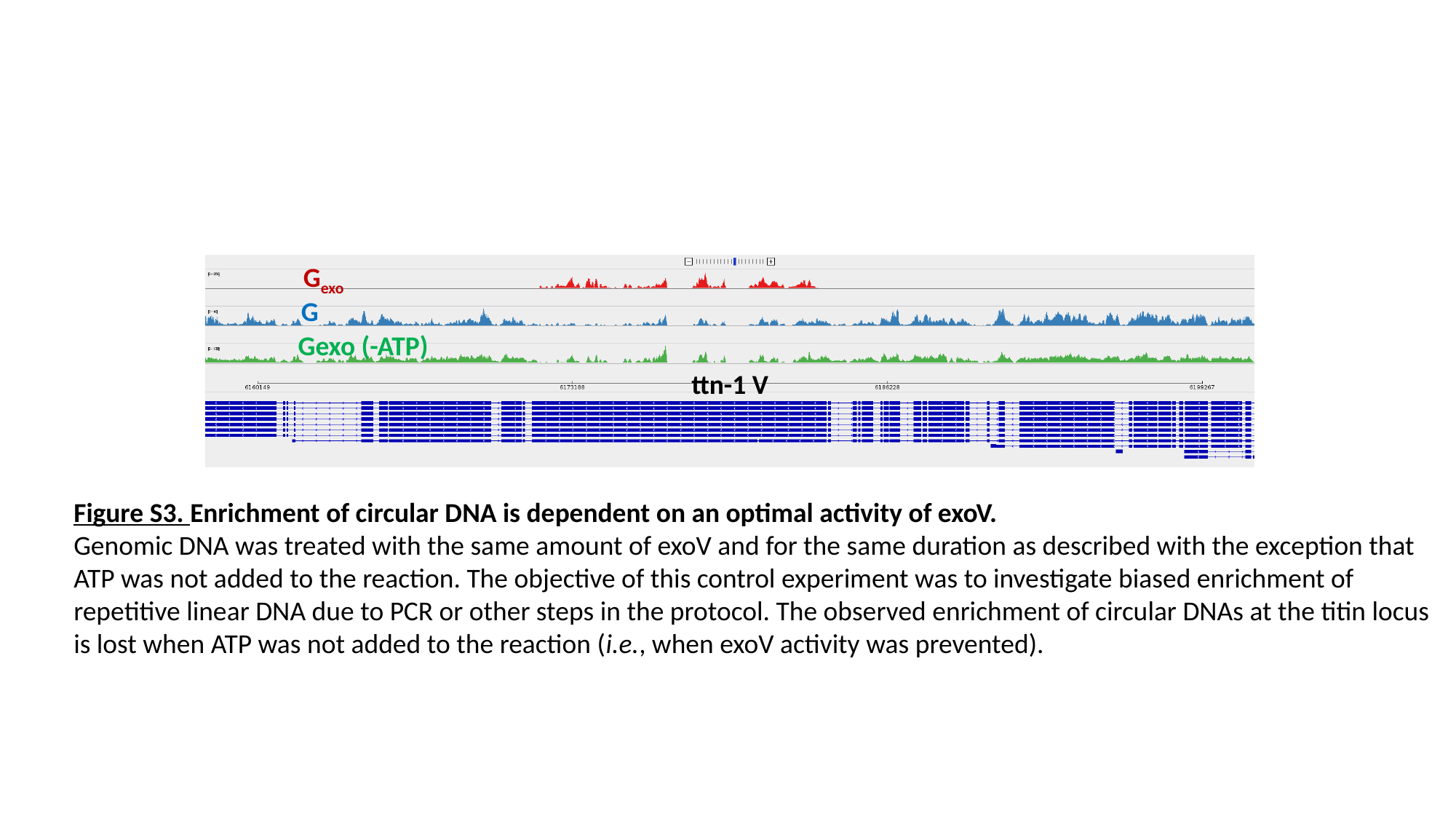

Gexo
G
Gexo (-ATP)
ttn-1 V
Figure S3. Enrichment of circular DNA is dependent on an optimal activity of exoV.
Genomic DNA was treated with the same amount of exoV and for the same duration as described with the exception that
ATP was not added to the reaction. The objective of this control experiment was to investigate biased enrichment of
repetitive linear DNA due to PCR or other steps in the protocol. The observed enrichment of circular DNAs at the titin locus
is lost when ATP was not added to the reaction (i.e., when exoV activity was prevented).
