# Supplementary material for: Intricate and Cell Type-Specific Populations of Endogenous Circular DNA (eccDNA) in Caenorhabditis elegans and Homo sapiens
Source: G3 (Bethesda). 2017 Aug 11;7(10):3295–303. doi: 10.1534/g3.117.300141 (PMC5633380; doi:10.1534/g3.117.300141)
Supplement: Supplementary file 4 [file 3295FigureS4.pptx]

## Slide 1
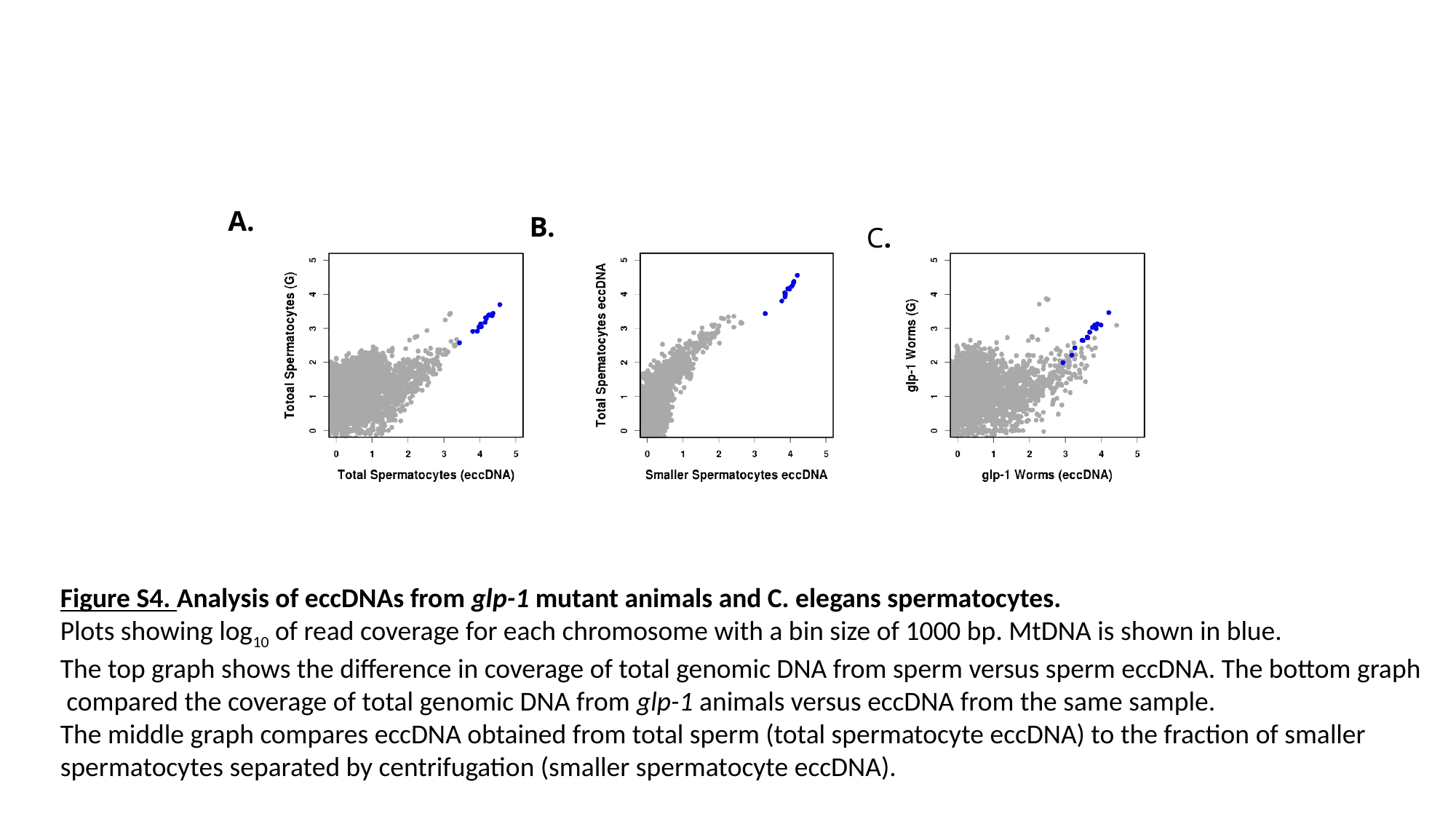

A.
B.
C.
Figure S4. Analysis of eccDNAs from glp-1 mutant animals and C. elegans spermatocytes.
Plots showing log10 of read coverage for each chromosome with a bin size of 1000 bp. MtDNA is shown in blue.
The top graph shows the difference in coverage of total genomic DNA from sperm versus sperm eccDNA. The bottom graph
 compared the coverage of total genomic DNA from glp-1 animals versus eccDNA from the same sample.
The middle graph compares eccDNA obtained from total sperm (total spermatocyte eccDNA) to the fraction of smaller
spermatocytes separated by centrifugation (smaller spermatocyte eccDNA).
