# Supplementary material for: Intricate and Cell Type-Specific Populations of Endogenous Circular DNA (eccDNA) in Caenorhabditis elegans and Homo sapiens
Source: G3 (Bethesda). 2017 Aug 11;7(10):3295–303. doi: 10.1534/g3.117.300141 (PMC5633380; doi:10.1534/g3.117.300141)
Supplement: Supplementary file 5 [file 3295FigureS5.pptx]

## Slide 1
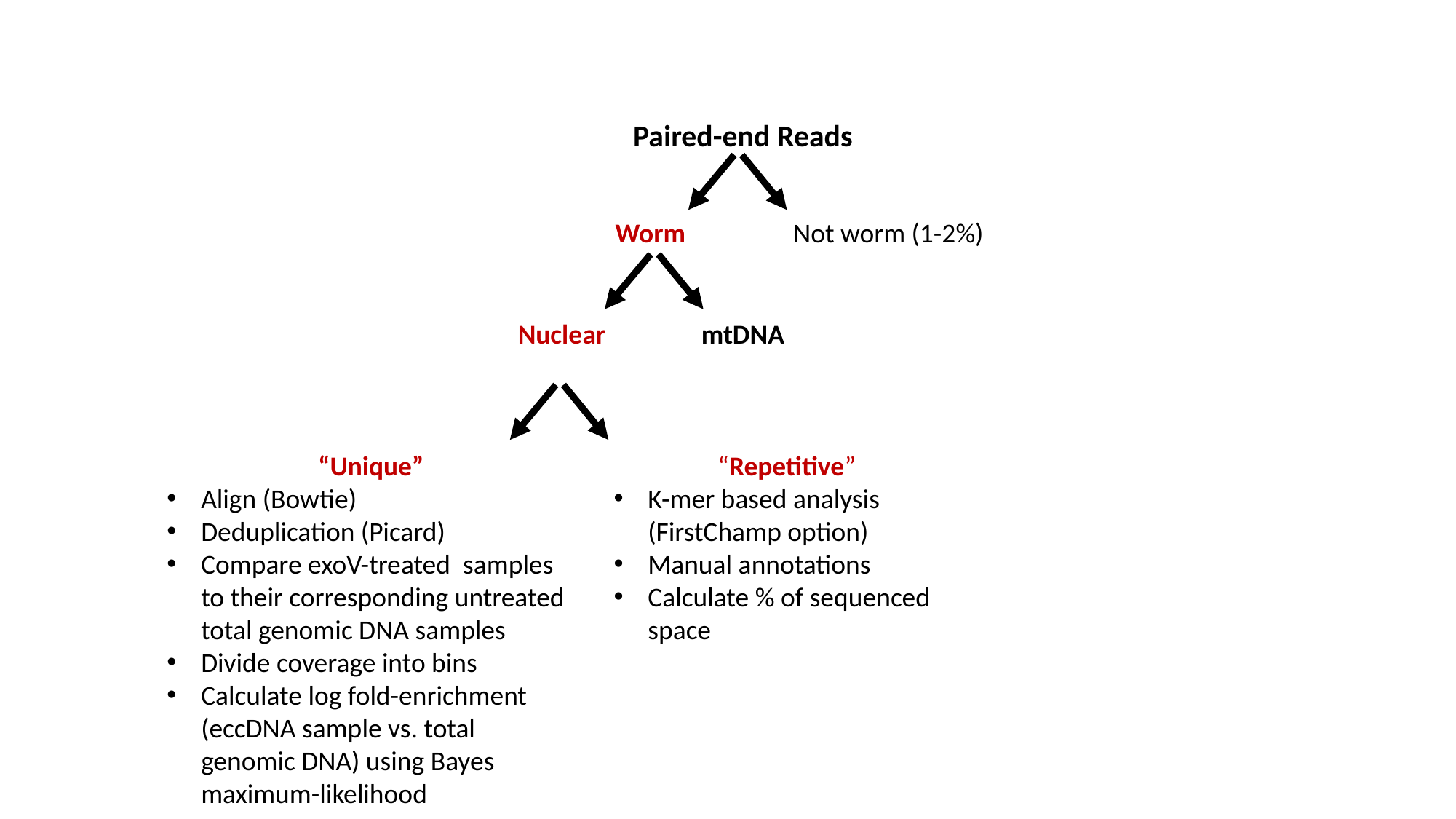

Paired-end Reads
Worm
Not worm (1-2%)
Nuclear
mtDNA
“Unique”
Align (Bowtie)
Deduplication (Picard)
Compare exoV-treated samples to their corresponding untreated total genomic DNA samples
Divide coverage into bins
Calculate log fold-enrichment (eccDNA sample vs. total genomic DNA) using Bayes maximum-likelihood
“Repetitive”
K-mer based analysis (FirstChamp option)
Manual annotations
Calculate % of sequenced space

## Slide 2
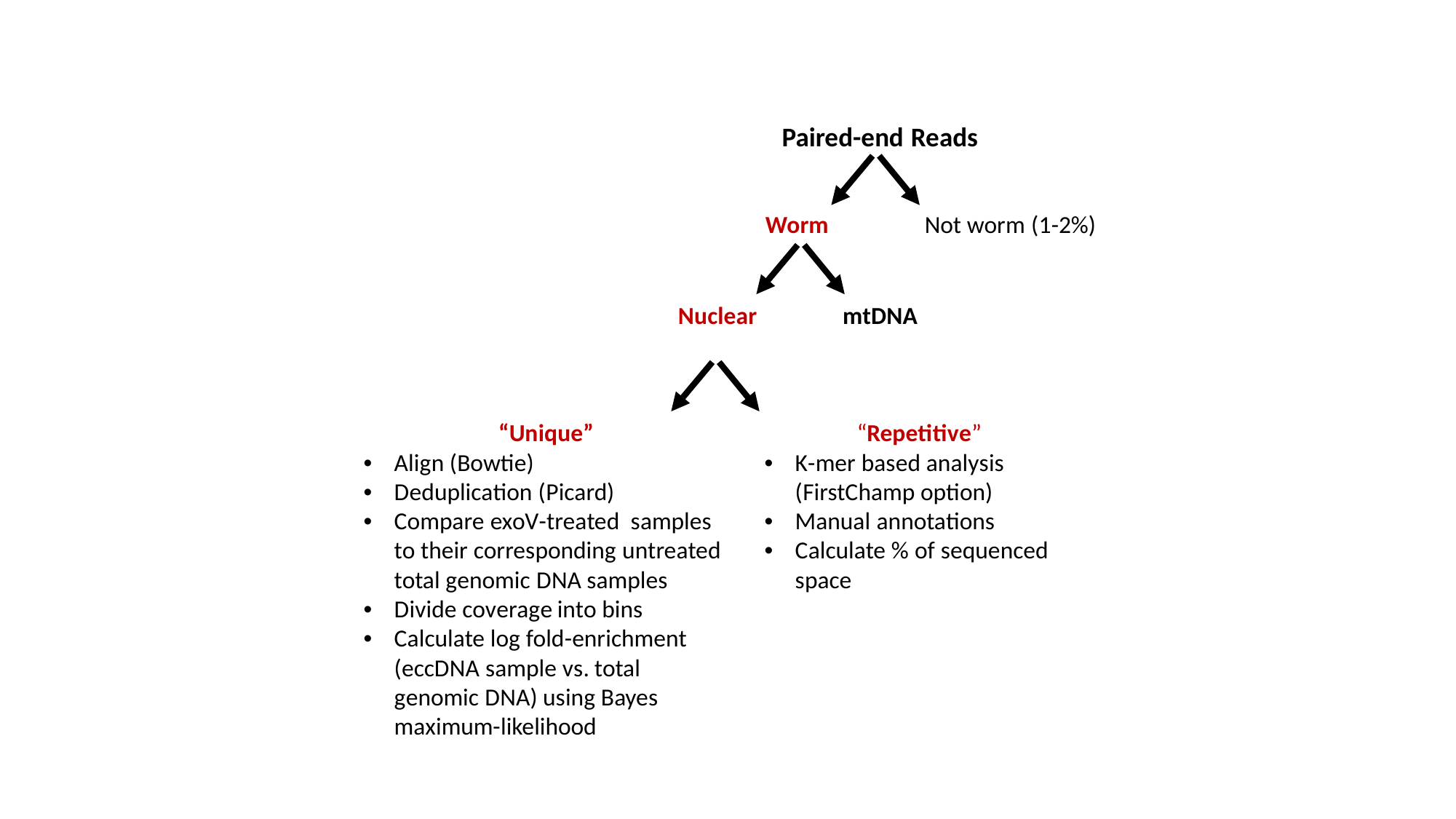

## Slide 3
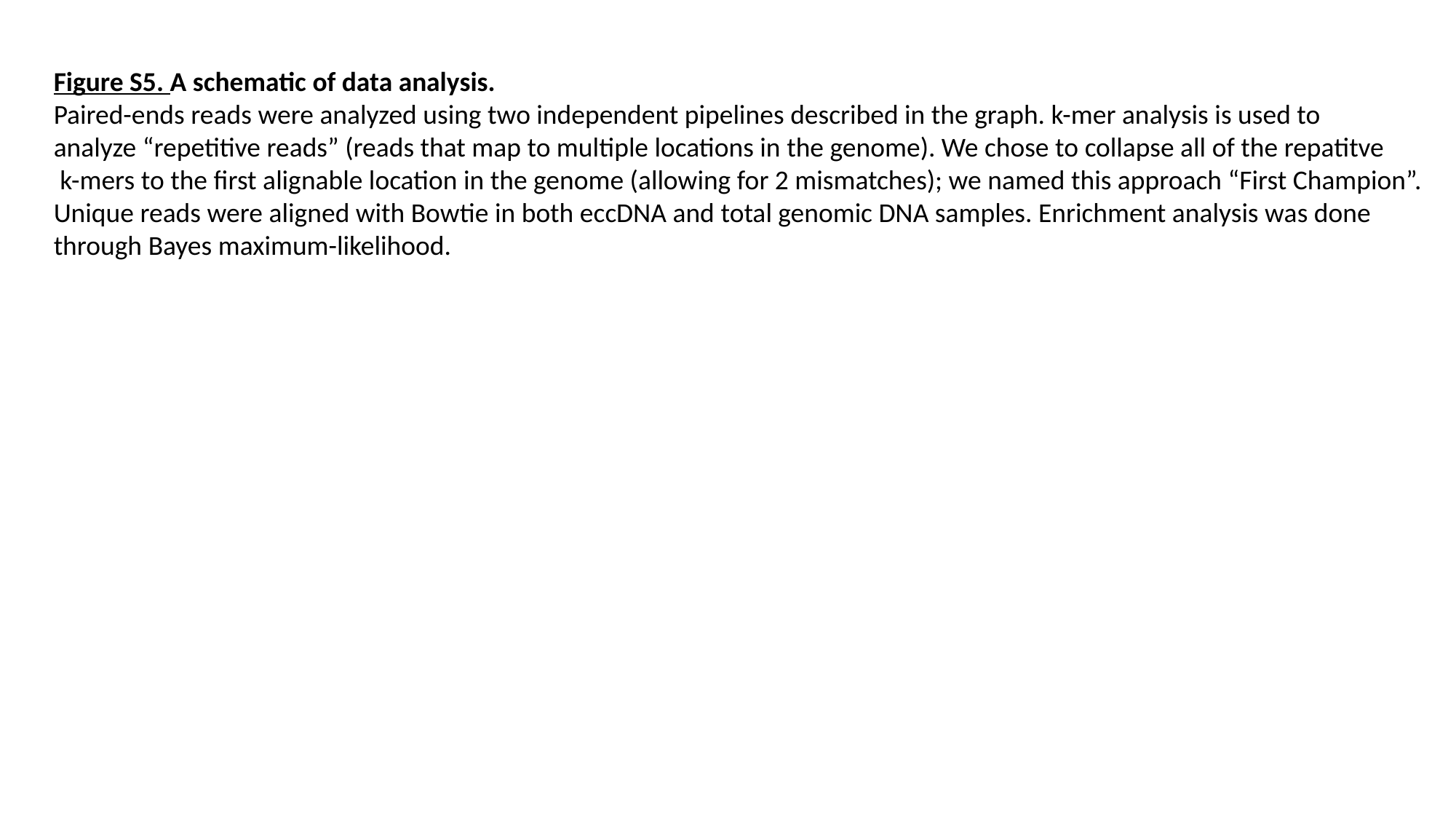

Figure S5. A schematic of data analysis.
Paired-ends reads were analyzed using two independent pipelines described in the graph. k-mer analysis is used to
analyze “repetitive reads” (reads that map to multiple locations in the genome). We chose to collapse all of the repatitve
 k-mers to the first alignable location in the genome (allowing for 2 mismatches); we named this approach “First Champion”.
Unique reads were aligned with Bowtie in both eccDNA and total genomic DNA samples. Enrichment analysis was done
through Bayes maximum-likelihood.
